# Effect of Placenta Previa on Preeclampsia

**DOI:** 10.1371/journal.pone.0146126

**Published:** 2016-01-05

**Authors:** Hao Ying, Yi Lu, Yi-Nuo Dong, De-Fen Wang

**Affiliations:** Department of Obstetrics, Shanghai First Maternity and Infant Hospital, Tongji University School of Medicine, Shanghai, China; Instituto de Ciências Biomédicas / Universidade de São Paulo—USP, BRAZIL

## Abstract

**Background:**

The correlation between gestational hypertension-preeclampsia (GH-PE) and placenta previa (PP) is controversial. Specifically, it is unknown whether placenta previa has any effect on the various types of preeclampsia (PE), and the role PP with concurrent placenta accreta (PA) play in the occurrence of GH-PE are not well understood.

**Objective:**

The aim of this study was to identify the effects of PP on GH, mild and severe preeclampsia (MPE and SPE), and early- and late-onset preeclampsia (EPE and LPE). Another aim of the study was to determine if concurrent PA impacts the relationship between PP and GH-PE.

**Methods:**

A retrospective single-center study of 1,058 patients having singleton pregnancies with PP was performed, and 2,116 pregnant women were randomly included as controls. These cases were collected from a tertiary hospital and met the inclusion criteria for the study. Clinical information, including PP and the gestational age at the onset of GH-PE were collected. Binary and multiple logistic regression analyses were conducted after the confounding variables were controlled to assess the effects of PP on different types of GH-PE.

**Results:**

There were 155 patients with GH-PE in the two groups. The incidences of GH-PE in the PP group and the control group were 2.5% (26/1058) and 6.1% (129/2116), respectively (P = 0.000). Binary and multiple regression analyses were conducted after controlling for confounding variables. Compared to the control group, in the PP group, the risk of GH-PE was reduced significantly by 78% (AOR: 0.216; 95% CI: 0.135–0.345); the risks of GH and PE were reduced by 55% (AOR: 0.451; 95% CI: 0.233–0.873) and 86% (AOR: 0.141; 95% CI: 0.073–0.271), respectively; the risks of MPE and SPE were reduced by 73% (AOR: 0.269; 95% CI: 0.087–0828) and 88% (AOR: 0.123; 95% CI: 0.055–0.279), respectively; and the risks of EPE and LPE were reduced by 95% (AOR: 0.047; 95% CI: 0.012–0.190) and 67% (AOR: 0.330; 95% CI: 0.153–0.715), respectively. The incidence of concurrent PA in women with PP was 5.86%; PP with PA did not significantly further reduce the incidence of GH-PE compared with PP without PA (1.64% vs. 2.51%, P>0.05). Binary logistic regression analyses were conducted after controlling for confounding variables, compared with the non-PP + GH-PE group, and the AOR of FGR in the non-PP + non-GH-PE group was 0.206 (0.124–0.342). Compared with the PP + GH-PE group, the AOR of FGR in the PP + non-GH-PE group was 0.430 (0.123–1.500).

**Conclusion:**

PP is not only associated with a significant reduction in the incidence of GH-PE, but also is associated with a reduction in incidence of various types of PE. Concurrent PA and PP do not show association with a reduction in incidence of GH-PE.

## Introduction

Gestational hypertension-preeclampsia (GH-PE) is a common pregnancy complication, with an incidence of 6–8% [1, 2]. In 1958, Bieniarz[3] reported that “there is no eclampsia in placenta previa cases, and on the other hand, in severe toxemia of late pregnancy low implantation of the placenta is met only exceptionally.” This claim aroused people's attention to the relationship between eclampsia and placenta previa (PP)[4, 5]. Although the exact cause of GH-PE is unclear, a lack of blood flow from the uterus to the placenta is always observed in cases of GH-PE[6], while the blood flow to the placenta in cases of PP is abundant [7]. Whether PP has a preventive effect on the occurrence or progression of GH-PE is therefore worthy of investigation.

There have been inconsistent findings with respect to the relationship between PE and PP: Ananth et al.[8] reported in 1997 that the presence of PP could reduce the incidence of PE by 50%. However, Hasegawa and Adam[4, 5], found no cases of PE present in women with PP, and Jelsema [9]reported that the presence of PP did not lower the incidence of PE. However, the sample sizes of these studies were small, with numbers of PP cases ranging from 200 to 300 [4, 5, 8, 10], and most of these studies did not take into consideration the influence of interfering factors such as BMI on the results of the study [8, 10]. What is more, to the best of our knowledge, no studies to date have investigated the effects of PP on various types of PE.

In the present study, we will not only discuss whether the presence of PP is associated with GH-PE, but we will also investigate the effects of PP on various types of GH-PE for the first time, which include GH, mild preeclampsia (MPE) and severe preeclampsia (SPE), as well as early- and late-onset preeclampsia (EPE and LPE, respectively).In addition, we will also discuss the effect of concurrent PP and placenta accreta (PA) on GH-PE.

## Materials and Methods

A retrospective single-center study was performed and a total of 62,327 women having singleton pregnancies with childbirth occurring between January 2010 and December 2014 were collected from a tertiary hospital (Shanghai First Maternity and Infant Hospital, Tongji University School of Medicine), among which 2,412 patients had abnormal position of the placenta. The exclusion criteria were: low-lying placenta; history of GH-PE; abnormal gestation history or birth history (stillbirth, placental abruption, fetal growth restriction [FGR], etc.);recurrent spontaneous abortion (RSA); use of specific medications during pregnancy (such as aspirin, low molecular weight heparin, glucocorticoids); lethal fetal malformation; complications during pregnancy (chronic hypertension, chronic kidney disease, type 2 diabetes, hyperthyroidism, hypothyroidism or autoimmune diseases). Based on the above screening criteria, a total of 1,058 women with PP were included in the study, and 2,116 normal pregnant women were randomly selected according to an individual case number, and included as controls (with the ratio between the experimental group and the control group being 1:2). All women in the PP group underwent a cesarean section. The Ethics Committee of Shanghai First Maternity and Infant Health Hospital, Tongji University School of Medicine approved the study. Since this was a retrospective study, consent was not obtained. All patient records/information were anonymized and de-identified prior to analysis.

### Diagnosis of PP[11, 12]

The diagnostic procedures for PP at our hospital include the following: the site of the placenta is assessed by abdominal ultrasound exam at 18–24 weeks of pregnancy; when PP is suspected, a transvaginal ultrasound exam is performed to determine if the lower edge of the placenta reaches or covers the internal os; a follow-up ultrasound exam is performed at 32 and 36 weeks of pregnancy; and the placenta location is confirmed right before delivery. If a cesarean section is performed, the relationship between the lower edge of the placenta and the internal os is assessed during surgery. On the other hand, if vaginal delivery is attempted, the distance between the rupture area of fetal membranes and the edge of the placenta is determined.

### Diagnosis of PA[13]

Placenta accreta has been defined as a condition wherein the placenta is adherent to the uterine wall and cannot be easily separated. This definition includes placenta accreta, increta, and percreta, based on histologic findings, or based on clinical findings if hysterectomy was not performed. The pathologic diagnosis of PA involves a hysterectomy performed due to hemorrhage. Pathological examination confirms that PA exists. In the PP group and control groups, a total of 390 patients needed manual removal of the placenta, and partial placental accrete was diagnosed in 65 patients whose placenta could not be completely separated. In all these 65 patients, 62 were in the PP group, and 3 were in the control group. In the 62 patients of the PP group, hysterectomy was performed in 5, and PA was confirmed through pathological examination, while 3 patients in the control group did not need hysterectomy.

### Diagnoses of GH and PE[14]

GH was defined as systolic blood pressure ≥140 mmHg and/or diastolic blood pressure ≥90mm Hg in a previously normotensive pregnant woman who is at ≥20 weeks of gestation and has no proteinuria or new signs of end-organ dysfunction. Preeclampsia was defined as the onset of hypertension after 20 weeks of gestation (systolic blood pressure, SBP ≥140mmHg and/or diastolic blood pressure, DBP ≥90mmHg) and proteinuria (≥0.3g in a 24-hour urine specimen or 1+ protein or more). Preeclampsia cases were considered severe if they had at least one of the following symptoms: SBP ≥160 mmHg or DBP ≥110 mmHg, proteinuria of 5g/24 hours, proteinuria of 3+ or more, oliguria, pulmonary edema, convulsions/eclampsia, or other abnormality. All other cases were considered mild. Early-onset preeclampsia is usually defined as preeclampsia that develops before 34 weeks of gestation, whereas late-onset preeclampsia develops at or after 34 weeks of gestation.

### Statistical analyses

Differences in GH-PE between women with and without PP, or with and without PA, were compared with descriptive and bivariate statistics using unpaired Student’s *t*-tests or a univariate general linear model for continuous variables and χ^2^tests or Fisher's exact test for categorical variables. Binary and multivariable logistic regression analyses were then conducted to analyze the effect of placenta previa on GH-PE while adjusting for potentially confounding variables, which include maternal age, gravidity, parity, prior cesarean section, BMI, and gestational age at delivery. Statistical significance was declared when the p-value was less than 0.05. The statistical analysis was performed using SPSS, version 19 Special Edition (SPSS, Inc., Chicago, IL, USA).

## Results

A total of 1,058 patients of PP met the inclusion criteria, and 2,116 patients were analyzed as controls. Compared to the control group, the PP group demonstrated older maternal age, higher maternal BMI, higher frequency of gravidity, higher ratio of previous delivery and higher ratio of prior cesarian section; fewer weeks of gestational age at delivery, and lower birth weight (Table 1).We performed statistical analysis using a univariate general linear model to correct for the influence of gestational age. The results showed that the two groups had no significant difference in birth weight.

**Table 1:** Comparison of clinical characteristics of pregnant women with and without placenta previa.

	PP (n = 1058)	Control (n = 2116)	F/χ^2^	P-value
Maternal age (years)	30.64 ± 4.70	29.27 ± 4.71	7.751	0.000
Gravidity				
≤2	417(39.4)	1158(54.7)	66.151	0.000
≥3	641 (60.6)	958 (45.3)		
MultiParity	224 (21.6)	245 (11.6)	51.550	0.000
Prior cesarean section	119(11.2)	107(5.1)	40.877	0.000
BMI(kg/m^2^)	21.48 ± 2.40	21.18 ± 2.33	3.412	0.000
Delivery (weeks)	36.59 ± 2.03	38.92 ± 1.79	-33.034	0.000
Birth weight (g)*	2890.68 ± 996.20	3265.50 ± 505.08	1.111	0.292
FGR	57(5.4)	119(5.6)	0.075	0.784

* Note: we performed statistical analysis using a univariate general linear model to correct for the influence of gestational age; the results showed that the two groups had no significant difference in birth weight.

GH-PE occurred in a total of 155 patients, and the incidences of GH-PE in the PP group and the control group were 2.5% (26/1058) and 6.1% (129/2116), respectively (P = 0.000) (Table 2). Binary logistic regression analyses were conducted after controlling for confounding variables. The incidence of GH-PE was significantly reduced by 78% in the PP group compared to the control group (AOR: 0.216; 95% CI: 0.135–0.345).The incidence of FGR in women with non-PP+ GH-PE was 20.9%,and in non-PP + non-GH-PE women was 4.6%, P = 0.000.The incidence of FGR in pregnant women with PP + GH-PE was 11.5%, and in pregnant women with PP+ non-GH-PE was 5.2% (P = 0.160). Binary logistic regression analyses were conducted after controlling for confounding variables; compared with the non-PP + GH-PE group, the AOR of FGR in the non-PP + non-GH-PE group was 0.206 (0.124–0.342). Compared with the PP + GH-PE group, the AOR of FGR in the PP + non-GH-PE group was 0.430 (0.123–1.500).

**Table 2:** Incidence of GH-PE in pregnant women with and without placenta previa.

	PP	Control	χ^2^	P-value	aOR [95% CI]
GH-PE	26 (2.5)	129 (6.1)	20.108	0.000	0.216 [0.135, 0.345]
GH	14 (1.3)	48 (2.3)	3.290	0.093	0.451 [0.233, 0.873]
PE	12 (1.1)	81 (3.8)	17.995	0.000	0.141 [0.073, 0.271]
Mild PE	4 (0.4)	36 (1.7)	9.925	0.002	0.269 [0.087, 0.828]
Severe PE	8 (0.8)	45 (2.1)	8.069	0.005	0.123[0.055, 0.279]
Early-onset	3 (0.3)	21 (1.0)	4.723	0.030	0.047 [0.012, 0.190]
Late-onset	9 (0.9)	60 (2.8)	13.067	0.000	0.330 [0.153, 0.715]

Note: Adjusted for maternal age, gravidity, parity, prior cesarean section, Brigand gestational age at delivery.

The incidence of both GH and PE was lower in the PP group than in the control group (1.3% vs. 2.3%, P = 0.093; 1.1% vs. 3.8%, P = 0.000). The analyses of the effects of PP on different types of PE showed that the incidence of both MPE and SPE was lower in the PP group than in the control group (0.4% vs. 1.7%, P = 0.002; 0.8% vs. 2.1%, P = 0.005). The analyses of the effects of PP on EPE and LPE demonstrated that the incidence of both EPE and LPE was lower in the PP group than in the control group (0.3% vs. 1.0%, P = 0.030; 0.9% vs. 2.9%, P = 0.000). Binary logistic regression analyses were conducted after controlling for the possible confounding variables, and the results indicated that: in the PP group, the incidences of GH and PE were reduced by 55% (AOR: 0.451; 95% CI: 0.233–0.873) and 86% (AOR: 0.141; 95% CI: 0.073–0.271), respectively; the incidences of MPE and SPE were reduced by 73% (AOR: 0.269; 95% CI: 0.087–0.828) and 88% (AOR: 0.123; 95% CI: 0.053–0.279), respectively; and the incidences of EPE and LPE were reduced by 95% (AOR: 0.047; 95% CI: 0.012–0.190) and 67% (AOR: 0.330; 95% CI: 0.153–0.715), respectively, compared to the control group.

The incidence of PA in the PP group and the control group was 5.86% (62/1058) and 0.15% (3/2116), respectively. The two groups were further divided into four groups according to PA status: non-PP + non-PA, non-PP + PA, PP + non-PA, and PP + PA; the incidence of GH-PE was 6.11% (129/2113), 0% (0/3), 2.51% (25/996) and 1.64% (1/62), respectively, in these four groups. Multivariable logistic regression analyses were conducted after controlling for confounding variables. Compared with the non-PP + non-PA group, the AOR of GH-PE in the PP + non-PA group and the PP + PA group was 0.396 (0.256–0.612) and 0.252 (0.035–1.833), respectively. Compared with the PP + non-PA group, the AOR of GH-PE in the PP + PA groupwas0.637 (0.085–4.778).

## Discussion

In the present study, we not only confirmed that the presence of PP is associated with a significant reduction in GH-PE, but we also determined that this lower-risk association of PP may also be associated with lower risk of both MPE and SPE and both EPE and LPE. However, we demonstrated that concurrent PA is not associated with further reduction in incidence of GH-PE.

Previous studies of PP focused mainly on its risk factors and the prevention and treatment of PP-related postpartum hemorrhage [15–17]. In recent years, an increasing number of researchers have begun to pay attention to the relationship between PP and PE, as well as the relationship between PP and FGR[4, 5]. According to Hasegawa and Adam [4, 5, 18, 19], the incidence of PE in their PP group was 0%, while it was 1.1% in our study. Was the incidence of PE in pregnant women with PP really 0, or was it just reduced? The answer might depend on the sample size of the studies. Either way, these results bring forth a new idea concerning the study of the pathogenesis of PE.

How is the presence of PP associated with a significant reduction in GH-PE? Here are some speculations: Perhaps with PP, the placenta implanted in the lower uterine segment gets a greater supply of blood and oxygen compared with the placenta implanted in the uterine body or fundus, so hypoxemia due to superficial implantation of placenta can be alleviated and vascular remodeling can be facilitated. Through this mechanism, the presence of PP may be associated with a reduction in GH-PE[10]. Alternatively, the trophoblasts attached in the lower uterine segment could infiltrate the helicine arteries more easily with PP[8]. In addition to the above speculations, there is an interesting phenomenon called superficial implantation of the placenta, which is one of the characteristics of PE[6, 20, 21]. In contrast, placenta previa and placenta accreta imply deep implantation of trophoblasts [7]. Thus, it seems that GH-PE and PP have opposite pathological mechanisms. Therefore, we speculate that deep infiltration of trophoblasts in PP can improve the blood supply and oxygenation of the placenta, so as to reduce GH-PE. This effect may be one of the reasons for reduced incidence of GH-PE in PP patients. Although the gestational age at delivery was earlier in the PP group than in the control group, binary regression analysis after the influence of gestational age was controlled for indicated that the risk of GH-PE in women with PP remained lower than in controls. This finding was consistent with the results of the study by Ananth in 1997[8]. Usually GH-PE increases the incidence of FGR, especially in cases of severe, early-onset GH-PE. This is related to impaired placental blood supply[22, 23]. Our results showed that in pregnant women without PP, GH-PE increased the incidence of FGR, a finding consistent with the literature[22, 23]. But surprisingly, in pregnant women with PP, the incidence of FGR in women with GH-PE was not significantly higher than in women without GH-PE. This finding is probably associated with increased placental blood supply in pregnant woman with PP.

Previous studies have not analyzed GH, MPE, and SPE separately. The present study is the first to demonstrate that the presence of PP is associated with a reduction in various types of GH-PE: the incidences of GH, MPE and SPE were reduced by 55% (AOR: 0.451; 95% CI: 0.233–0.873), 73% (AOR: 0.269; 95% CI: 0.087–0.828) and 88% (AOR: 0.123; 95% CI: 0.055–0.279), respectively. This study is also the first to report that PP not only is associated with a reduction in EPE, but is also associated with a reduction in LPE. It is commonly believed that the pathogenesis of EPE and LPE has some differences[24–27]. Specifically, EPE results from superficial implantation of the placenta, which means it is a disease of placental origin (based on the superficial implantation theory), whereas LPE is mainly caused by maternal factors, such as maternal physiological overreaction to inflammatory changes[26, 28–31]. After the influence of gestational age at delivery was excluded with statistical methods, the incidence of LPE was still lower in the PP group than in the control group, which suggests that in addition to maternal factors, placental factors perhaps also play a role in the occurrence of LPE.

As mentioned above, the reduction in the risk of GH-PE in the PP group might be attributable to deep implantation of trophoblasts. Indeed, trophoblasts will infiltrate more deeply when concurrent PA occurs [32, 33]. Corthorn et al. demonstrated that the expression of cytokines that facilitate the infiltration of trophoblasts such as kallikrein and endothelial nitric oxide synthase was significantly increased in women with PA compared to in women with PE [34]. Therefore, we speculated that when concurrent PP and PA occurs, the trend toward a reduction in the incidence of GH-PE might be even more significant. Unfortunately, the results of this study showed that PP with concurrent PA is not associated with further reduction in the incidence of GH-PE. This pattern suggests that multiple factors play a role in GH-PE, and that decreased infiltration of trophoblasts and superficial implantation of the placenta resulting from its incomplete vascular remodeling are just two possible factors [24].

There are several limitations to our study. First, although the sample size of the PP group was 1,058 patients, which to the best of our knowledge was the largest sample size for this kind of study, when the patients were further divided based on MPE and SPE, or EPE and LPE, the number of cases of each type was reduced significantly, to single digits. This was particularly the case when the patients were further divided into a PA group and non-PA group. Second, the diagnosis of PA in this study was not entirely based on pathologic examination; most of the patients were diagnosed based on clinical information. PA was diagnosed when manual removal of the placenta was conducted after delivery. Subjectivity is often involved in clinical diagnosis, which affects the assessment of the relationship between PA and GH-PE. Third, although we speculate that the vascular supply in the placenta previa group might have been better than in the control group, we didn’t have any data on uterine artery Doppler investigations. Lastly because the number of GH-PE patients in the PP group was less than 10, we did not further analyze the relationship between different types of PP (complete, partial or marginal) and GH-PE.

PP is associated with a significant reduction in not only GH-PE, but also various types of PE. Concurrent placental accreta may not be associated with further reduction in PE.

## Supporting Information

S1 Dataset All of the raw data and calculation results in the SPSS sav file. (SAV)

## Abbreviations

GH: gestational hypertension
PE: preeclampsia
MPE: mild preeclampsia
SPE: severe preeclampsia
EPE: early-onset preeclampsia
LPE: late-onset preeclampsia
PP: placenta previa
PA: placenta acreta
FGR: fetal growth retardation
BMI: body mass index
AOR: adjusted odds ratio
CI: confidence interval
